# Fourier-plane wavefront and SLM aberration characterization via iterative scanning of beam deflector segments

**DOI:** 10.1038/s41598-025-32903-0

**Published:** 2025-12-23

**Authors:** Antoni J. Wojcik, Dilawer Singh, Ayan Rakshit, Hannah J. Joyce, Timothy D. Wilkinson

**Affiliations:** Electrical Engineering Division, Department of Engineering, University of Cambridge, 9 JJ Thomson Avenue, Cambridge, CB3 0FA UK

**Keywords:** Engineering, Optics and photonics, Physics

## Abstract

This study presents a method for characterizing the effective incident wavefront in the Fourier plane, eliminating the need to decouple measurement and application. The amplitude and phase of this wavefront are cumulatively affected by the incident beam, the curvature of the backplane, and the defocus introduced by the Fourier lens. Using an iterative camera-in-the-loop approach, this method displays square patches of beam deflection phase ramps on the spatial light modulators (SLMs), and the resultant spots in the Fourier plane are positionally controlled. The parameters of each patch are used to reconstruct the phase and amplitude profiles of the wavefront. These profiles are used in hologram computation to correct for SLM aberrations and improve image quality. The proposed method has been experimentally verified to work with off-axis and on-axis optical systems, showing the versatility of the approach.

Spatial light modulators (SLMs) are devices capable of modulating either phase or amplitude of incident light. In this work, we consider phase-only modulation by nematic liquid crystal on silicon (LCoS) SLMs, but the presented ideas can be extended to amplitude modulation and other phase modulation devices. The patterns displayed on such devices, called *holograms*, modulate the incident coherent wavefronts to recreate the encoded light distributions, called *images*, through optical diffraction and interference. The process of calculating these patterns is termed computer-generated holography (CGH).

CGH by default regards SLMs as perfectly flat devices with ideal pixel responses. However, in practice, manufacturing-induced strain introduces curvature in the backplane and cover glass^1,2^, causing the thickness of the liquid crystal (LC) layer to vary across the device and distorting the wavefront phase. Furthermore, SLMs are typically designed for planar wavefronts at normal incidence (Fig. 1(a)), but this configuration requires beam splitters, introducing unwanted reflections. Off-axis systems (Fig. 1(b)) avoid these reflections, but cause light to interact with the LC medium at an angle, leading to unpredictable phase accumulation. Additionally, the incident wavefront itself may have non-uniform phase and amplitude profiles, for example due to the Gaussian intensity distribution typical of laser sources or imperfections in the optical system. Neglecting any of the mentioned aspects leads to unwanted aberrations^3^, which in severe cases cause the reconstructed images to be unrecognizable.

The curvature of the SLM backplane can be measured with standard optical flatness measurement procedures such as the Michelson interferometer^4^ and derivative approaches^5,6^. These methods are typically limited to on-axis optical configurations and have several disadvantages, such as reliance on precise alignment of the SLM with a reference surface of sub-wavelength flatness. Alternatively, Shack-Hartmann wavefront sensors can be used to characterize aberrations but necessitate reconfiguring the optical system, making the process less versatile and more time-consuming. However, any change in SLM alignment, such as slight tip/tilt between measurement and application, can alter the observed aberrations.

**Fig. 1:**
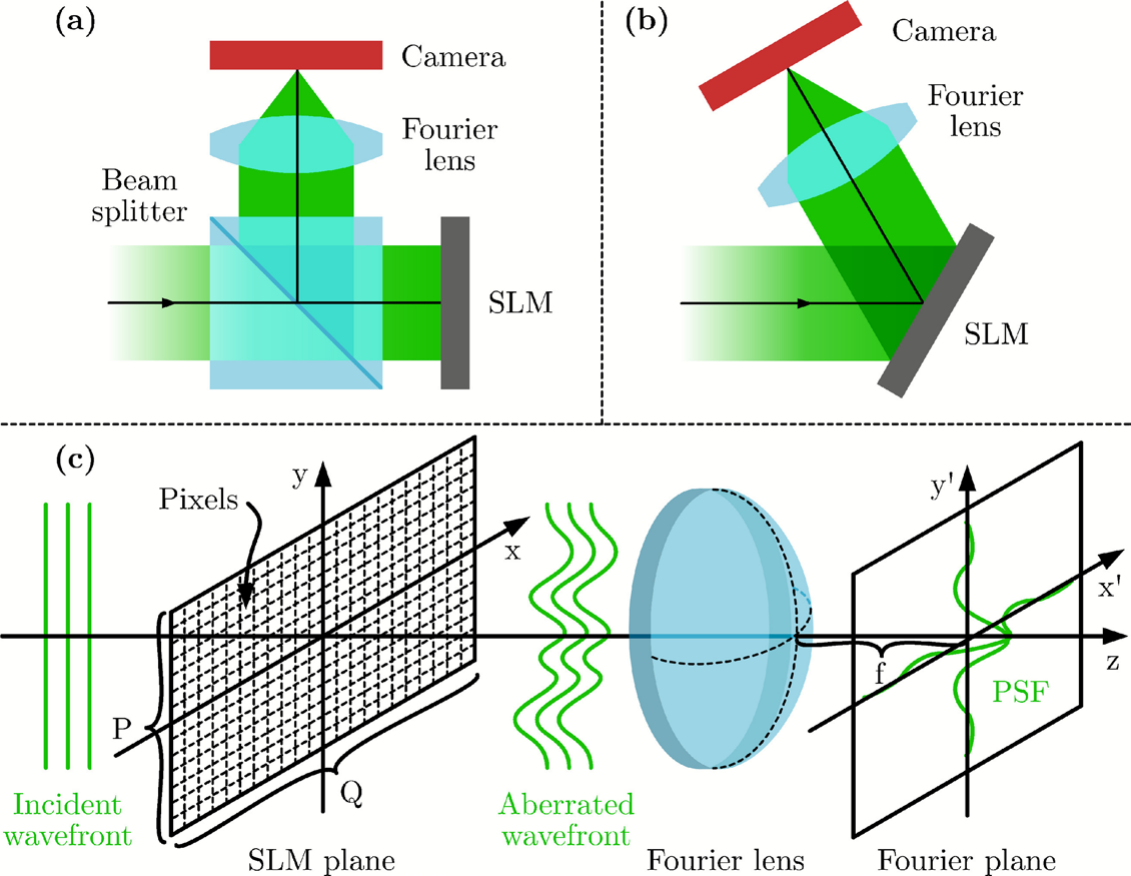
(**a**), (**b**) Schematic diagrams of on-axis and off-axis holographic optical systems, respectively. (**c**) Fourier optical system involving an SLM. Commonly used SLMs are reflective, but here the optical axis was “unfolded” for clarity. A wavefront of unknown profile in amplitude and phase is incident on the SLM, and the reflected wavefront becomes aberrated because of the non-uniform LC layer. This affects the shape of the PSF in the Fourier plane.

Another common approach to characterize aberrations is to fit Zernike polynomials by minimizing the size of the observed point spread function (PSF) in the Fourier plane^3,7,8^. This involves scanning and fitting the Zernike coefficients so that the PSF, observed after reflecting from the SLM and passing through a Fourier lens (Fig. 1(c)), approximates the ideal aberration-free case. Typically, the lowest ($\sim$15) Zernike modes suffice for effective correction. However, these methods also have several limitations. Zernike polynomials are orthogonal on a circular domain, while SLMs are rectangular, requiring either restricting analysis to the central circular area of the SLM while neglecting the rest, or implmenting an ad hoc approximation where only the central rectangular portions of the polynomials are used. This issue can be addressed by using rectangular Zernike modes, which increases computational complexity^9^. Additionally, the PSF may be smaller than the camera pixel size in systems with high numerical apertures (depends on the size of the aperture and the focal length used), reducing accuracy. Also, since most incident light is focused into the PSF, it requires a high dynamic range, which can be difficult or time-consuming to capture with a camera. Finally, these approaches do not directly measure the incident amplitude distribution.

Our approach realizes that all aberration sources in a Fourier optical system affect the formed images collectively, and can be characterized as the phase and amplitude of a single effective complex field at the SLM. The method divides the SLM into square segments and sequentially displays phase-ramp beam deflector patterns in each segment. The resulting spot positions in the Fourier plane, together with their total power, provide enough information to reconstruct the complex reflected wavefront. This approach is conceptually similar to the operation of Shack-Hartmann sensors, with the key difference being that the same optical setup can be used to first characterize and then test holographic projections using the same field lens instead of swapping in a microlens array. This eliminates the need to reconfigure the optics after the measurement. The method is suited to operate in both configurations shown in Figs. 1(a) and (b), as it is agnostic to the orientation of the SLM relative to the camera.

## Methods

### Motivation

Fourier optical systems can be modeled by unfolding them along the principal axis, as shown in Fig. 1(c). In an ideal system, the diffraction of the field *E*(*x*, *y*) from the SLM plane at spatial coordinates (*x*, *y*) to the field *U*(*u*, *v*) at the focal plane at spatial frequencies (*u*, *v*) is given by the Fraunhofer diffraction integral^10^ (neglecting constant amplitude and phase factors):

1 $$\begin{aligned} U(u, v) = \mathscr {F}\left\{ E(x,y)\right\} \equiv \iint _{\mathbb {R}^2} E(x, y) e^{-2 \pi j (x u + y v)} \!\textrm{d}x \!\textrm{d}y \end{aligned}$$

where $\mathscr {F}$ represents the Fourier transform. We refer to the focal plane of the lens as the Fourier plane, and for a lens of focal length *f*, its spatial coordinates can be described as $(x', y') = (f \lambda u, f \lambda v)$ using the paraxial approximation, where $\lambda$ is the wavelength of light. However, the (*u*, *v*) coordinates are used in this section as the derived equations are easier to follow. In case of phase-only modulation used here, the complex field *E*(*x*, *y*) may be expanded as:

2 $$\begin{aligned} E(x, y) = A_\text {wav}(x,y) e^{j( \phi _\text {wav}(x,y) + \phi (x,y))} \end{aligned}$$

where we define $A_\text {wav}(x,y) = P(x,y) A_\text {inc}(x,y)$ and $\phi _\text {wav}(x,y) = \phi _\text {inc}(x,y) + \phi _\text {aberr}(x,y) + \phi _\text {lens}(x,y)$ for simplicity. *P*(*x*, *y*) is the rectangular aperture of the SLM. $A_\text {inc}(x,y)$ and $\phi _\text {inc}(x,y)$ represent the amplitude and phase of the incident wavefront, respectively, $\phi _\text {aberr}(x,y)$ is the aberrated phase due to the non-flatness of the SLM, $\phi _\text {lens}(x,y)$ is the defocus between the reconstruction plane and the focal plane of the lens, and $\phi (x,y)$ is the phase displayed at the SLM.

In practice, reflections from the inactive area of the SLM outside *P*(*x*, *y*) and from glass surfaces of optical elements, termed *undiffracted light*, mix with the original wavefront creating complicated, non-ideal interferences. However, these fields are static and typically slowly varying, forming a pattern around $(u,v) = (0,0)$, which we refer to as the *zeroth (diffraction) order*.

**Fig. 2:**
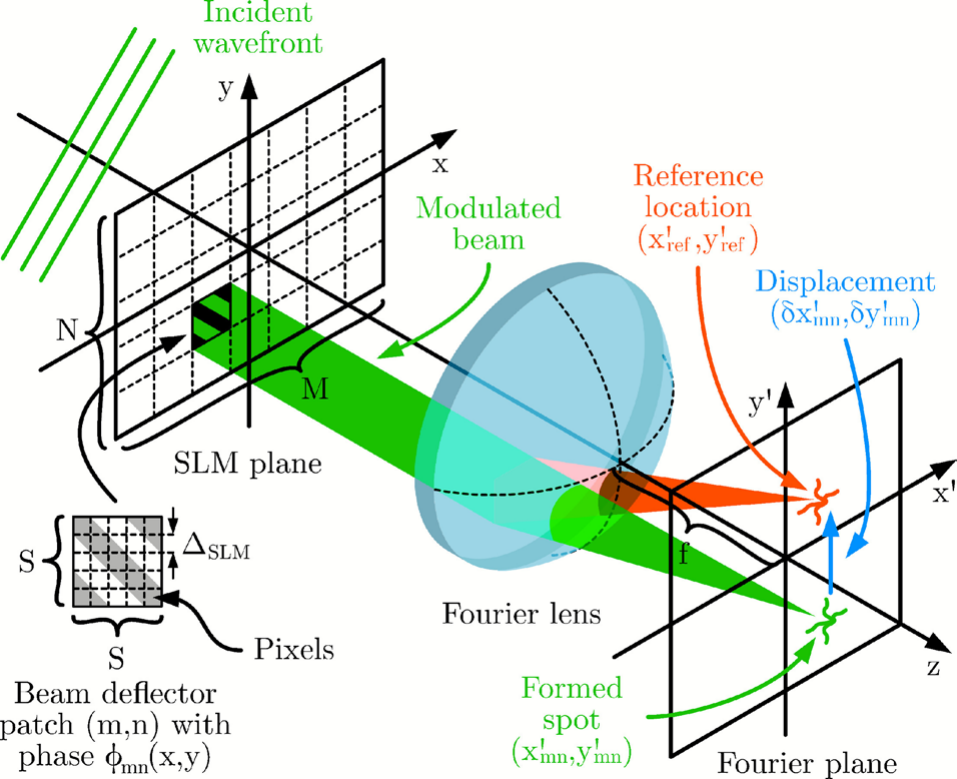
An illustration of our method of displaying a beam deflector patch (*m*, *n*) at the SLM, which projects a point in the Fourier plane (green) at position $(x'_{mn}, y'_{mn})$ (where $(x', y') = (f \lambda u, f \lambda v)$). This position differs from the reference location $(x'_\text {ref}, y'_\text {ref})$ by $(\delta x'_{mn}, \delta y'_{mn})$ because of aberrations and misalignment of the optical system.

This approach divides the surface of the SLM of size $P \times Q$ pixels (see Fig. 1(c)) into an array $M \times N$ of square patches of side length *S* in pixels, as shown in Fig. 2. *S* can be chosen as the greatest common divisor $S = \gcd {(P, Q)}$ or its integer factor. In this way $M = P / S$ and $N = Q / S$, so the tiled patches fill the entire SLM. The physical side length of the patch is $a = S \Delta _\text {SLM}$, where $\Delta _\text {SLM}$ is the pixel pitch of the SLM. At any given time, one patch (*m*, *n*) is displaying the following phase ramp deflecting light to spatial frequencies $(u_0, v_0)$, away from the zeroth order, while all other patches are set to zero:

3 $$\begin{aligned} \begin{aligned} \phi _{mn}(x, y)&= P_\text {patch}(x - x_{mn},y - y_{mn}) \, 2\pi \left[ (x - x_{mn}) u_0 + (y - y_{mn}) v_0 \right] \\ \text {where } P_\text {patch}(x,y)&= 1 \text { if } x, y \in \left[ -a/2, a/2\right] ,\ 0 \text { otherwise} \end{aligned} \end{aligned}$$

where $P_\text {patch}(x,y)$ is a square aperture of the patch centered at $(x_{mn}, y_{mn})$ coordinates. In optimal conditions where $A_\text {wav}(x,y) = P(x,y)$ and $\phi _\text {wav}(x,y) = 0$, Eq. (1) gives:

4 $$\begin{aligned} \begin{aligned}&U(u,v) = U_0 {\textrm{sinc}{ \left[ a (u - u_0) \right] }} {\textrm{sinc}{ \left[ a (v - v_0) \right] }} e^{ -2 \pi j \left[ x_{mn} (u - u_0) + y_{mn} (v - v_0) \right] }\\ \text {where }&U_0 = a^2 \text { and } {\textrm{sinc}{ (x) }} = \sin {(\pi x )}/(\pi x) \end{aligned} \end{aligned}$$

This equation neglects the contribution of unmodulated light reflected from outside the patch in the zeroth order for simplicity. The camera can only detect intensity $I(u,v) = {\left| {U(u,v)} \right| }^2$, so:

5 $$\begin{aligned} I(u,v) = I_0 {\textrm{sinc}{ \left[ a (u - u_0) \right] }}^2 {\textrm{sinc}{ \left[ a (v - v_0) \right] }}^2, \text { where } I_0 = U_0^2 \end{aligned}$$

Eq. (5) shows that each patch acts as a beam deflector, diverting light to a point at $(u_0, v_0)$ with a two-dimensional sinc envelope.

Experimentally, $A_\text {wav}(x,y)$ and $\phi _\text {wav}(x,y)$ will have a different and unknown form, which will affect the observed *U*(*u*, *v*). Both quantities can be approximated as varying smoothly across the SLM, as is the case for a Gaussian beam illumination and typical backplane curvatures. This means that if *S*, consequently *a*, are sufficiently small, $A_\text {wav}(x,y)$ can be treated as a constant $A_{mn}$ and $\phi _\text {wav}(x,y)$ approximated to have constant gradient $\textbf{g}_{mn}$ over the area of the patch:

6 $$\begin{aligned} A_\text {wav}(x,y)&\approx A_\text {wav}(x_{mn}, y_{mn}) = A_{mn}\end{aligned}$$

7 $$\begin{aligned} \phi _\text {wav}(x,y)&\approx \textbf{g}_{mn} \cdot \textbf{x} = 2\pi (x \alpha _{mn} + y \beta _{mn}) \end{aligned}$$

where $(\alpha _{mn}, \beta _{mn})$ in the gradient $\textbf{g}_{mn} = (2\pi \alpha _{mn}, 2\pi \beta _{mn})$ corresponds to the displacement of the formed spot (see Eq. (8)), $\textbf{x} = (x,y)$, and “$\cdot$” denotes the dot product. The gradients $\textbf{g}_{mn}$ can be thought of as the mean gradient $\textbf{g}(x,y) = \nabla \phi _\text {wav}(x,y)$ over the patch (*m*, *n*): $\textbf{g}_{mn} = {\left\langle {\textbf{g}(x,y)} \right\rangle }_{mn}$. Thus, by following the same reasoning as above, the observed intensity becomes:

8 $$\begin{aligned} I(u,v) \approx I_0 {\left| {A_\text {wav}(x_{mn}, y_{mn})} \right| }^2 {\textrm{sinc}{ \left[ a (u - u_0 - \alpha _{mn}) \right] }}^2 {\textrm{sinc}{ \left[ a (v - v_0 - \beta _{mn}) \right] }}^2 \end{aligned}$$

Based on this equation, by displaying different beam deflector patches with equal $(u_0, v_0)$, we expect the observed point to have the same general form but be shifted by a certain $(\alpha _{mn}, \beta _{mn})$ relative to $(u_0, v_0)$ for each patch (*m*, *n*), and the Gaussian illumination to scale the intensity by ${\left| {A_\text {wav}(x_{mn}, y_{mn})} \right| }^2$. This information is used to recover $A_\text {wav}(x,y)$ and $\phi _\text {wav}(x,y)$.

### Implementation

The SLM enables control over patterns in the *first (diffraction) order* defined by $(u, v) \in [-0.5/\Delta _\text {SLM}, 0.5/\Delta _\text {SLM}]$^10^. A small value of $(u_0, v_0) \approx (0.1 / \Delta _\text {SLM}, 0.1 / \Delta _\text {SLM})$ is chosen so that the deflected point is away from the zeroth order and can be clearly identified but is still close to the center. The following procedure is implemented (see Fig. 2 for clarification):

1. **Reference point initialization:** Display the patch at the center of the SLM $(x_\text {ref}, y_\text {ref}) = (0, 0)$. Capture the formed image and select a region of interest (ROI) around the observed point. Then use Otsu’s image thresholding method^11^ to select the point and calculate its center of mass (COM): $(x'_\text {ref}, y'_\text {ref}) \leftarrow \int _\text {point} \textbf{x}' I(x',y') \!\textrm{d}x' \!\textrm{d}y' / \int _\text {point} I(x',y') \!\textrm{d}x' \!\textrm{d}y'$, where $\textbf{x}' = (x', y')$. This will serve as the reference to find the phase gradients of other patches.
  $\triangleright$ Ideally, this COM would lie at coordinates $(x'_\text {ref}, y'_\text {ref}) = (f \lambda u_0, f \lambda v_0)$, but experimentally, the camera might be offset from the center of the Fourier plane and its sensor may be misaligned with the plane. This potential misalignment of the camera is why the COM of the reference point is measured experimentally instead of being set manually.
2. **Patch scanning:** Iterate over all patches (*m*, *n*). For each, find the best fit for the gradient $\tilde{\textbf{g}}_{mn} = 2\pi (\tilde{\alpha }_{mn}, \tilde{\beta }_{mn})$ that cancels out the aberration-related gradient ($\tilde{\textbf{g}}_{mn} +\textbf{g}_{mn} = 0$), with the following camera-in-the-loop (CITL) approach:
  - Display the phase from Eq. (3) and calculate the COM of the spot in the ROI at coordinates $(x'_{mn}, y'_{mn})$. From this, calculate the separation vector from the reference point: $\delta \textbf{x}'_{mn} = (\delta x'_{mn}, \delta y'_{mn}) \leftarrow (x'_{mn} - x'_\text {ref}, y'_{mn} - y'_\text {ref})$.
  - Set $\tilde{\textbf{g}}_{mn} = (2\pi \tilde{\alpha }_{mn}, 2\pi \tilde{\beta }_{mn}) \leftarrow (2\pi \delta x'_{mn} / (f \lambda ), 2\pi \delta y'_{mn} / (f \lambda ))$. Display the phase:
    9 $$\begin{aligned} \phi _{mn}(x,y) = P_\text {patch}(x - x_{mn},y - y_{mn}) \, 2\pi [x (u_0 - \tilde{\alpha }_{mn}) + y (v_0 - \tilde{\beta }_{mn})] \end{aligned}$$
    Afterwards, measure the new COM of the spot in the ROI and calculate $\delta \textbf{x}'_{mn}$.
    $\triangleright$ This causes the spot to shift according to Eq. (8), and sets a root-finding problem $\delta \textbf{x}'_{mn} = (0, 0)$ which means $(\tilde{\alpha }_{mn}+\alpha _{mn}, \tilde{\beta }_{mn} + \beta_{mn}) = (0, 0) \rightarrow \tilde{\textbf{g}}_{mn} = -\textbf{g}_{mn}$, where $\delta \textbf{x}'_{mn}$ is treated as an unknown function of $\tilde{\textbf{g}}_{mn}$.
  - Use one step of the Brent’s root-finding method^12^ to update $\tilde{\textbf{g}}_{mn}$, which solves $\delta \textbf{x}'_{mn} = (0, 0)$ based on the previous values of $\tilde{\textbf{g}}_{mn}$ and $\delta \textbf{x}'_{mn}$. Display the phase in Eq. (9) and measure the new COM of the spot in the ROI and calculate $\delta \textbf{x}'_{mn}$.
    $\triangleright$ Brent’s method was chosen because it achieved the fastest convergence in experiments. Secant method was also tested to work successfully.
  - Repeat step 2(c) until $\delta r_{mn}' = |\delta \textbf{x}'_{mn}| = \sqrt{|\delta x'_{mn}|^2 + |\delta y'_{mn}|^2} \le \varepsilon$, where $\varepsilon$ is a tolerance parameter, or a maximum number of iterations $N_I$ is reached.
  - Find the total power $P_{mn}$ within the ROI as $P_{mn} = \int _\text {ROI} I(x',y') \!\textrm{d}x' \!\textrm{d}y'$. Calculate the local amplitude as $A_{mn} \leftarrow \sqrt{P_{mn}}$.
    $\triangleright$ In an ideal scenario, the obtained $\tilde{\textbf{g}}_{mn}$ could approximate $-\textbf{g}_{mn}$ in a single step 2(b). However, even a slight optical misalignment of the lens or the camera away from the focal plane will make this formula imprecise, so in this way, the subsequent steps are more effective. The final $\tilde{\textbf{g}}_{mn}$ is a good approximation to $-\textbf{g}_{mn}$ and both terms are further used interchangeably as $\textbf{g}_{mn} = -\tilde{\textbf{g}}_{mn}$.
3. **Wavefront reconstruction:** Once all $\textbf{g}_{mn}$ and $A_{mn}$ are found, $\phi _\text {wav}(x,y)$ and $A_\text {wav}(x,y)$ are calculated across all the pixels of the SLM. $A_\text {wav}(x,y)$ is approximated by first normalizing $A_{mn} \leftarrow A_{mn} / \max (A)$, and then applying cubic interpolation across the patches. Recovery of $\phi _\text {wav}(x,y)$ from $\textbf{g}_{mn}$ involves two main steps (see SI, “Wavefront reconstruction” for details). First, the discrete gradients are interpolated to obtain a smooth gradient field $\textbf{g}(x,y)$ across the SLM, using a bilinear interpolation scheme that ensures continuity between patches. Second, the phase $\phi _\text {wav}(x,y)$ is recovered by solving the Poisson equation $\nabla ^2 \phi _\text {wav} = \nabla \cdot \textbf{g}$, which is performed in the Fourier domain as:
  10 $$\begin{aligned} \phi _\text {wav}(x,y) = - \mathscr {F}^{-1} \left\{ \frac{\mathscr {F}\left[ \nabla \cdot \textbf{g}\right] (u,v)}{(2\pi )^2 (u^2 + v^2)} \cdot \textbf{1}_{(u,v)\ne (0,0)} \right\} . \end{aligned}$$
  $\textbf{1}_{(u,v)\ne (0,0)}$ is an indicator function that is zero at the origin to avoid division by zero, effectively setting the mean phase to zero. This approach yields a smooth phase profile consistent with the measured gradients, while avoiding error accumulation over large areas. This equation can be implemented using Fast Fourier Transforms (FFTs).

**Fig. 3:**
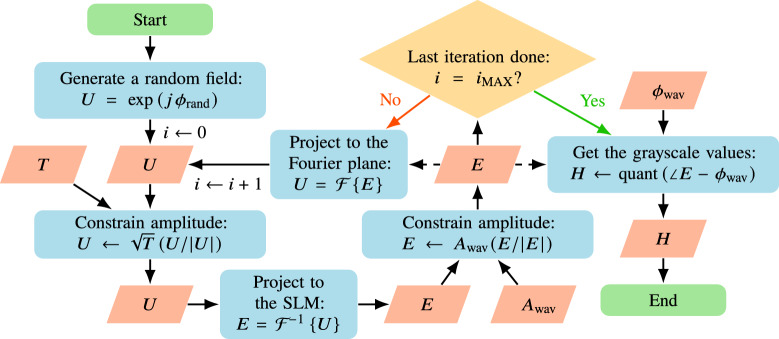
Gerchberg-Saxton algorithm taking into account the measured amplitude and phase to generate the hologram *H*. Here $\phi _\text {rand}$ has values $\in [-\pi , \pi )$, $\angle E$ represents the phase of the complex field *E*, and $\textrm{quant}()$ is a function that quantizes continuous values to integer grayscale values that can be displayed at the SLM.

Once the reflected complex wavefront is characterized, the results are included in the hologram generation process. For this, a modified implementation of the Gerchberg-Saxton (GS) algorithm^13^ is used to generate Fourier holograms, presented in Fig. 3. The method incorporates the measured $A_\text {wav}(x,y)$ and $\phi _\text {wav}(x,y)$ and fixes the target intensity *T*(*u*, *v*) in the Fourier plane (corrected for the sinc envelope from the square SLM pixels) to generate the optimized hologram *H*(*x*, *y*) quantized to grayscale values displayed on the SLM. The combined phase accumulated on the SLM is: $\phi (x,y) + \phi _\text {wav}(x,y) - \phi _\text {wav}(x,y) = \phi (x,y)$, so the aberrations are removed. In the calculations, the fields on the SLM are zero-padded to ensure that no aliasing occurs when calculating the fields in the Fourier plane and are cropped when projecting back to the SLM plane^10^.

## Results

**Fig. 4:**
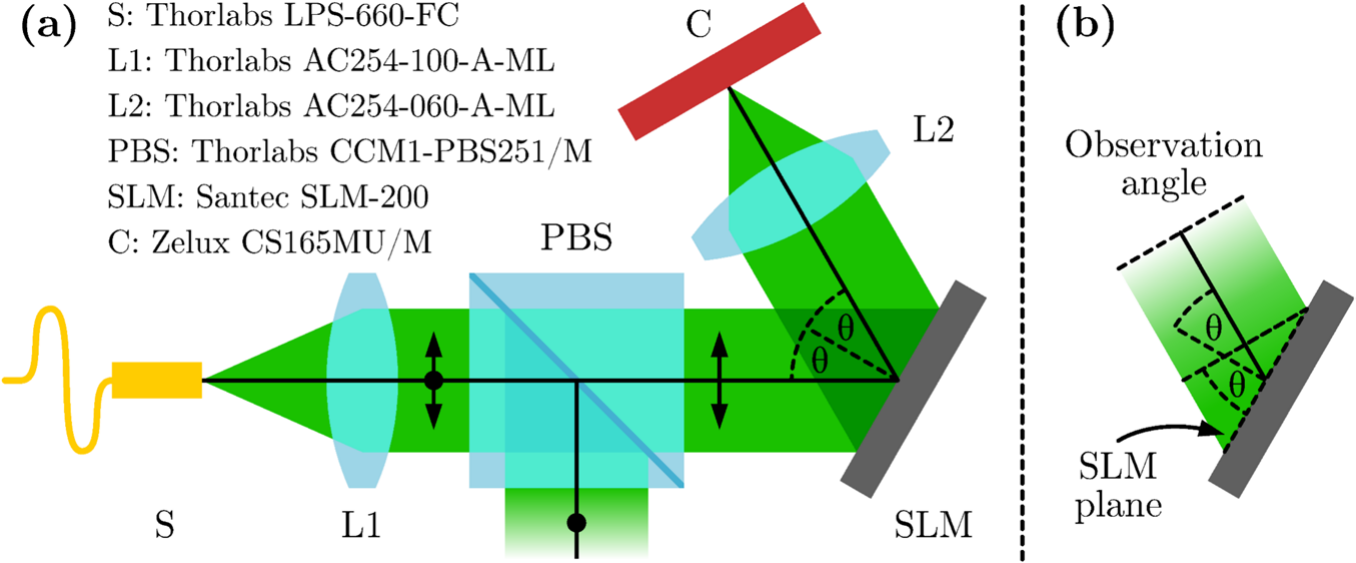
(**a**) Schematic diagram of the experimental optical system. The single-mode fiber laser source, S, emits light at wavelength $\lambda = {658}\,\text {nm}$. The polarizing beam splitter, PBS, is used to select the polarization of light along the LC director of the SLM. Lens L1 is used to collimate the beam, and Fourier lens L2 focuses the light onto the camera, C. (**b**) Compressed projection of the SLM plane onto the observation direction (other optical elements are omitted for clarity).

The proposed wavefront characterization method was tested with the off-axis optical system shown in Fig. 4(a), with the SLM at a tilt angle of $\theta \approx {25}^{\circ }$. This angle is unusually large for SLM operation, but it was chosen to demonstrate the adaptability of our approach. Fourier lens L2 of focal length $f = {6}\,\text {cm}$ was used in conjunction with a laser source of wavelength $\lambda = {658}\,\text {nm}$. This was to ensure that the full range of the first diffraction order fell within the camera sensor of size $1440 \times 1080$ pixels, with pitch $\Delta _\text {cam} = {3.45}\,{\upmu }\textrm{m}$. The deflected points were sufficiently separated from the zeroth order, so no blocking filter was required.

The SLM has size of $P \times Q = 1920 \times 1200$ pixels, with pitch $\Delta _\text {SLM} = {8}\,{\upmu }\textrm{m}$. Thus, a factor of $\gcd {(1920, 1200)} = 240$ was chosen for the patch size, $S = 120$, which gives $M \times N = 16 \times 10$ patches in total. The following parameters were used: $(u_0, v_0) = (0.1 / \Delta _\text {SLM}, -0.1 / \Delta _\text {SLM})$, $N_I = 6$, and $\varepsilon = {0.1}$ pixels, which were sufficient for most patches in the experiments. The results presented in this section have the *y*-axis oriented downwards.

**Fig. 5:**
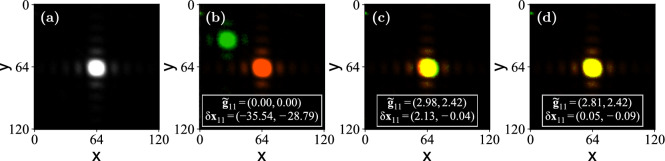
Point shifting process for the patch (1, 1) in an example measurement. The colors were modified in post-processing. (a) ROI with the reference point. (b)-(d) ROI with the observed point shown in green and the reference point superimposed in red: (b) visible after the first iteration when $\tilde{\textbf{g}}_{11} = (0, 0)$, (c) after the second iteration (step 2(b)), and (d) after the third (step 2(c)). The yellow area indicates the overlap between the two spots. The exact values of the used gradients $\tilde{\textbf{g}}_{11}$ in units of rad/patch and the observed separation $\delta \textbf{x}_{11}$ in units of rad/pixel are showed at the bottom of the images.

Figure 5 shows an example convergence of the position for the patch (1, 1). Fig. 5(a) shows the reference spot and Fig. 5(b) the resulting spot for the patch (1, 1) with the initial gradient, $\tilde{\textbf{g}}_{11} = (0,0)$, after step 2(a) of the procedure described in “Implementation”. The CITL process converges in only three iterations. The step 2(b) of the procedure in the second iteration moves the point nearly to the reference position, and step 2(c) only adds a marginal improvement in the third iteration. In Fig. 5(c) small red and green portions can be seen around the yellow overlap area, indicating that $\delta \textbf{x}_{11}'$ is not optimized, but in Fig. 5(d), the spots overlap completely, as reflected in small values of $\delta \textbf{x}_{11}'$. The final $\delta r_{11}' = 0.09 < \varepsilon$, so the loop is stopped before the number of iterations, $N_I$, is reached. The quick convergence in this case was due to the good approximation in step 2(b) in the plane where the measurement was taken, but more iterations could be necessary for different planes.

**Fig. 6:**
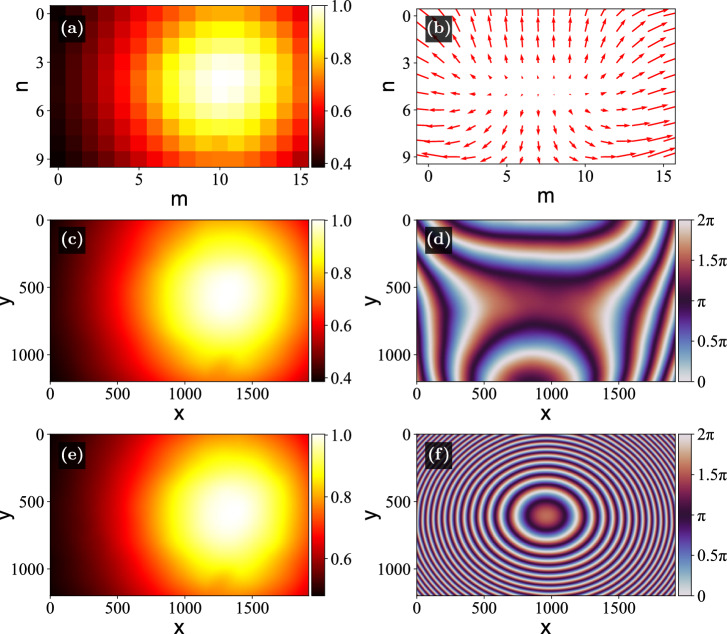
(**a**), (**b**) Measured normalized amplitudes and gradients at each patch. (**c**), (**d**) Amplitude and phase across the area of the SLM recovered from the patch data in (**a**) and (**b**). (**e**), (**f**) Recovered amplitude and phase for a displaced camera.

To demonstrate versatility of this approach, the experiments were conducted for two different camera placements: with the sensor in the focal plane, and shifted by $\Delta z \approx {1}\,\hbox {mm}$ away from the focal plane. Figs. 6(a) and (b) show the measured values of $A_{mn}$ and $\textbf{g}_{mn}$ for the first case, and Figs. 6(c) and (d) present the reconstructed $A_\text {wav}(x,y)$ and $\phi _\text {wav}(x,y)$. The amplitude distribution appears Gaussian, as expected from a single-mode fiber laser. Figs. 6(e) and (f) show the same results but for the second configuration. The amplitude distribution is again Gaussian and similar to that of Fig. 6(c). The small discrepancies can be attributed to the coarseness of the used array (set by the chosen $S=120$), which may violate the assumptions of flatness and constant gradients, and optical noise that appear in the ROIs of the different measurements that affect the measured $P_{mn}$. The phase in Fig. 6(f), calculated with the camera sensor displaced by $\Delta z \approx {1}\,\hbox {mm}$, shows a pronounced parabolic component compared to Fig. 6(d), where the sensor lies in the focal plane of the lens. This is caused by a significant defocus aberration $\phi _\text {lens}(x,y)$ introduced by this displacement, which is known to be quadratic^10^. This confirms that the method “inferred” that a compensating defocus phase term needs to be present in the wavefront to counteract $\phi _\text {lens}(x,y)$, in effect bringing the image in focus despite the offset from the focal plane of the Fourier lens.

**Fig. 7:**
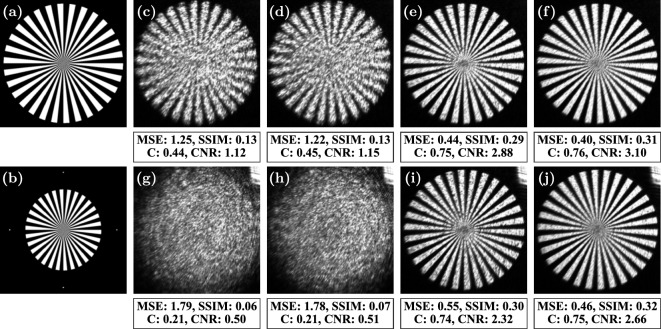
(**a**) Target spoke pattern. (**b**) Pre-compressed target pattern, shown together with reference points used for the affine transformation. (**c**)-(**f**) Reconstructions without any correction, with only amplitude correction, with only phase correction, with amplitude and phase corrections, respectively, with the camera placed at the focal plane. (**g**)-(**j**) Reconstructions for the same parameters as (**c**)-(**f**), but at the displaced camera. In (**g**)-(**j**), the zeroth order is visible in the top right corners of the images. The values of MSE, SSIM, C, and CNR are shown at the bottom of each corresponding image.

To verify that the wavefront characterization is correct and usable, a spoke wheel target image shown in Fig. 7(a) was used in Fourier holography. The holograms displayed on the SLM were generated using the modified GS algorithm (see “Implementation” and Fig. 3). The experiments were performed in the exact same configurations as used in the characterization experiments. Due to the tilt of the SLM relative to the camera, the projected plane is compressed along the $x'$-axis by $\cos {\theta }$ (see Fig. 4(b)). This causes the image to appear elongated by $1 / \cos {\theta }$ along the $x'$-axis. To compensate for this, the target pattern was compressed by $\cos {{25}^{\circ }} = 0.91$ of its original width, as shown in Fig. 7(b). Accurate analysis of the observed images was done through an affine transformation, based on four registration points placed along the edges of the pattern in the target image. Four different holograms were calculated, with $i_\text {max} = 300$ iterations of the modified GS algorithm for each, to observe the particular effects of the measured wavefront on image reconstruction: without any correction, with only the amplitude correction, with only the phase correction, and with both amplitude and phase corrections. The images were captured with a camera and aligned using the affine transform calculated with reference to the target image. Then, four metrics were used to measure the change in their quality: mean squared error (MSE), contrast (C), contrast-to-noise ratio (CNR) and the structural similarity index measure (SSIM)^14^. The first three are defined as follows:

11 $$\begin{aligned} \textrm{MSE} = {\left\langle {{\left| {\tfrac{I}{{\left\langle {I} \right\rangle }} - \tfrac{T}{{\left\langle {T} \right\rangle }}} \right| }^2} \right\rangle } \qquad \text {and} \qquad \textrm{C} = \frac{{\left\langle {I_B} \right\rangle } - {\left\langle {I_D} \right\rangle }}{{\left\langle {I_B} \right\rangle } + {\left\langle {I_D} \right\rangle }} \qquad \text {and} \qquad \textrm{CNR} = \frac{{\left\langle {I_B} \right\rangle } - {\left\langle {I_D} \right\rangle }}{\sqrt{\sigma _B^2 + \sigma _D^2}} \end{aligned}$$

where ${\left\langle {X} \right\rangle }$ denotes the mean value of *X* over all pixels, ${\left\langle {I_B} \right\rangle }$ and ${\left\langle {I_D} \right\rangle }$ are the mean pixel values of the bright and dark regions determined by comparison to the target image, respectively, and $\sigma _B$ and $\sigma _D$ are the corresponding standard deviations.

The results for both experimental arrangements are shown in Figs. 7(c)-(f) and 7(g)-(j), respectively. The four metrics show a systematic improvement as the measured wavefront properties are introduced in the CGH process: C, CNR and SSIM go up, and the MSE is reduced. Correcting for only the amplitude is not sufficient to obtain recognizable images under the present aberrations, but combining amplitude and phase correction does improve the uniformity of the observed images. This is because the value of $A_\text {wav}(x,y)$ within different areas (*x*, *y*) of the SLM determines how much power different spatial frequencies contribute to the formed image. An improved estimate of these values enables the CGH algorithm to better use these frequencies in the reconstruction process. However, to see these improvements, the aberrations determined by $\phi _\text {wav}(x,y)$ must first be corrected. The reconstruction of the image in the case of the displaced camera appears slightly worse, confirmed by worse metric values, because it is more difficult to correct small relative errors when stronger aberrations are present. Using a finer array of the patches might improve the results, but because the SLM is pixelated while the fields are continuous, even an ideal wavefront characterization could not fully correct for the aberrations. Nevertheless, the results prove that our method significantly improves the quality of the formed images in Fourier holography.

## Discussion

The results shown in the previous section prove that the proposed method accurately measures the effective incident wavefront and corrects aberrations in the focal plane. The method inherently accounts for aberrations $\phi _\text {lens} (x,y)$ arising from any displacement of the camera from the true focal plane. This ensures that the formed image is in focus in the sensor plane, which is advantageous for Fourier-plane applications such as holographic lithography. The method also measures the incident amplitude, which is important in CGH because Fourier holography effectively treats SLM pixels as frequency components of the encoded image. The otherwise assumed flat wavefront incorrectly assigns equal weight to all spatial frequencies, which is not generally true. This is the case for commonly used Gaussian beams produced by single-mode lasers unless the beam is greatly expanded during preparation, wasting a large portion of their power.

The maximum spatial frequency that can be resolved with this method in either $A_\text {wav}(x,y)$ or $\phi _\text {wav}(x,y)$ is limited by the Nyquist frequency of patch sampling, 1/(2*a*)^15^. Variations above this limit remain local to each patch, distorting the ideal sinc-like spot and biasing estimates of the local $\textbf{g}_{mn}$ or $A_{mn}$, which reduces the reconstruction accuracy of both fields (see SI, “Reconstruction errors”). The shape of the spot can practically indicate whether such aberrations are significant: if it maintains the expected sinc profile, the patch size *a* is sufficient; otherwise, it should be reduced. However, *a* cannot be made arbitrarily small, as very small patches produce sinc spots that are too broad and faint (see Eq. (8)) to be reliably captured due to camera sensitivity, shot noise, or sensor size. Fortunately, high-frequency contributions, such as those caused by dust, are usually localized and have minimal impact on $\textbf{g}_{mn}$. Although the method cannot resolve them, this is acceptable in most applications, as such aberrations have little effect on the focal plane due to the Fourier relationship between the SLM and the focal plane.

Another important consideration in the characterization process is the ROI size, which must be large enough to capture all deflected spot positions. If a spot for the patch (*m*, *n*) appears outside the ROI, the CITL algorithm fails for that patch, and then $\textbf{g}_{mn}$ and $A_{mn}$ have incorrect values, typically $A_{mn} \approx 0$. However, the ROI cannot be too large because other orders of the beam deflector or the zeroth order can enter the ROI, thus making the calculations of the COM incorrect and leading to wrong results. This problem could potentially be avoided by using more advanced computer vision tracking algorithms. Similarly, the laser power and the saturation time must be chosen so that the spots are visible but do not over-saturate the camera pixels. Otherwise, the measured values of $P_{mn}$ and consequently $A_{mn}$ and $A_\text {wav}(x,y)$ will be incorrect, and the phase measurement will be affected.

Even without such characterization errors, the method is inherently biased toward global tip/tilt (see SI, “Reconstruction errors”), which affects the measured $\phi _\text {wav}(x,y)$ (SI, Fig. S5). This is also the case for interferometric methods and occurs because all observed points, including the reference, may be shifted. Subtracting the mean gradient from $\textbf{g}_{mn}$ partially mitigates but does not remove this bias. In practice, the remaining tip/tilt only shifts the image in the focal plane and can be compensated by pre-shifting the target pattern. Absolute tip/tilt correction requires an objective reference point at the ideal location $(f \lambda u_0, f \lambda v_0)$, which in practice can be determined relative to the zeroth-order spot that defines the optical axis and is generally unaffected by aberrations.

While this text focuses on the off-axis configuration, the proposed method is not limited to such systems. We also tested it in on-axis Fourier systems, which are simpler to characterize since they are the intended SLM configuration. We tested different laser wavelengths (${405}\,\hbox {nm}$ and ${635}\,\,\hbox {nm}$), different SLMs (Santec SLM-250 and Holoeye LUNA) and different lenses (see SI, “Other experiments”). The results of these experiments are consistent with those presented in “Results”. The comparable performance across different wavelengths and hardware configurations validates the versatility of our approach. This is expected because the wavelength only changes the rate of phase variation, but not its overall shape, as the underlying physics remains unchanged. Likewise, most LCoS SLMs share similar fabrication processes and therefore exhibit similar types of aberrations.

The main time-limiting bottleneck in our current implementation is the SLM LC response. For the Santec device, this leads to an average measurement time of 12.5 min, whereas Holoeye LUNA requires only 50 s due to its much faster response. Although the Santec SLM is specified with an LC response time of ${200}\,{\upmu }\hbox {s}$, we observed that a delay of about 1.5 s was needed between displaying a pattern and capturing the image (only ${100}\,\hbox {ms}$ for LUNA). Otherwise, the phase appeared unstable, likely due to communication latency. Each patch typically requires three iterations of the beam deflectors, resulting in about 4.5 s per patch on a $16 \times 10$ grid. The method itself is not inherently slow and could be further accelerated by parallelizing the scan over multiple patches, provided that interference between the projected spots is avoided. However, even with such improvements, real-time correction of rapidly varying aberrations such as those induced by air turbulence remains unlikely for nematic LCoS SLMs using this technique, but could be within reach for much faster digital micromirror devices.

**Fig. 8:**
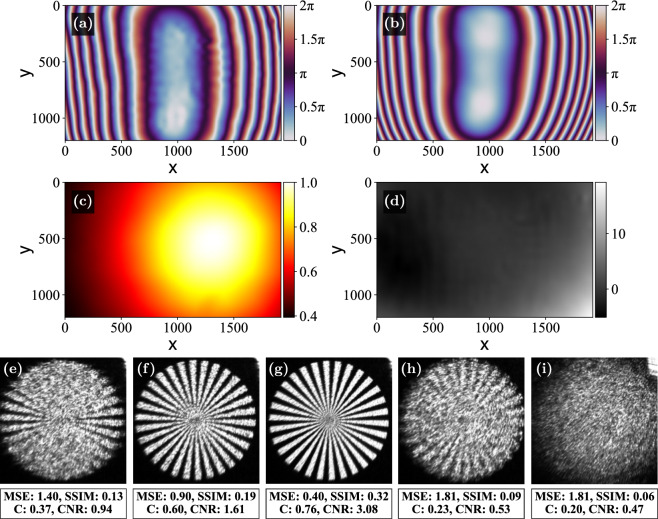
(**a**) Phase correction pattern provided by the SLM manufacturer, obtained using an interferometer. (**b**), (**c**) Phase correction pattern and measured amplitude obtained using the proposed method. (**d**) Unwrapped phase of the difference between phases in (**a**) and (**b**). (**e**)-(**g**) Reconstructed images generated without any correction, using the phase from (**a**), and using the phase from (**b**) and amplitude from (**d**), respectively. (**h**) and (**i**) Observed images using the phase from (**a**) observed in the two experimental configurations examined in “Results”.

To see how the method compares with an alternative technique, we compared holograms generated using the proposed method with the ones generated using the phase measurement obtained using a Shack-Hartmann wavefront sensor, provided by the SLM’s manufacturer^16^ and shown in Fig. 8(a). The provided phase contains a quadratic component, which slightly shifts the Fourier plane, so the comparison required moving the camera away from the focal plane of the lens to find the best plane where the holograms form focused images. This process is tedious, and determining the optimal camera position is difficult even when using translation stages, highlighting a limitation of methods that require reconfiguring the optical system for measurement. This is not the case for the proposed approach, which finds the corrected wavefront for the exact position of the camera.

The phase and amplitude for the optimal location are shown in Figs. 8(b) and (c). The phase in Fig. 8(b) was measured at the camera position where the images formed using the phase correction pattern from (a) appear most in focus. This position was determined by first applying the correction from (a), then adjusting the camera to minimize aberrations, and finally calculating the correction at that plane using our method. The smooth discrepancy between the measured and provided phases shown in Fig. 8(d) is likely caused by the aforementioned tip/tilt bias, a slight defocus because the positions of the planes are not exactly matched, or by the fact that the provided phase was measured in an on-axis configuration. As explained earlier, tilting the SLM after measurement may affect aberrations as the incident light interacts with the LC layer of the SLM, the glass cover, and the backplane at an angle. Fig. 8(e) shows the observed uncorrected image, and Figs. 8(f) and (g) show the comparison between the corresponding corrected images. The tested quality metrics indicate that the proposed method achieves better results. The new method also has the advantage that it uses the measured $A_\text {wav}(x,y)$ in the CGH process, while the other method can only assume a flat wavefront. This leads to a smoother image in Fig. 8(g). To complete the comparison, Figs. 8(h) and (i) show the images obtained when the provided phase is used in the two experimental arrangements described in “Results” rather than in its matched configuration. In both cases, the quality metrics are worse than if no correction is used at all. This is not a completely fair comparison, as the provided phase was measured in a different plane, but it does indicate the versatility of our approach.

The results above show again that our method works reliably in both off-axis and on-axis systems. In contrast, the interferometric characterization methods are difficult to adapt to off-axis systems because the SLM-reflected beam must be recombined with a reference beam, which is much easier in an on-axis geometry such as Fig. 1(a).

A similar approach, treating the SLM as a Shack-Hartmann wavefront sensor, was previously proposed in^17^. However, that implementation relied on fitting Zernike polynomials to the captured images, whereas the method presented here employs an automated iterative CITL process. As a result, it is not constrained to low-order Zernike modes, which are also ill-suited for the rectangular aperture of the SLM. Furthermore, we provide a formal and comprehensive procedure for recovering both the phase and amplitude profiles from the measured gradients and intensities, thereby extending and reinforcing the concept.

The method also resembles those presented in^18,19^, where small square beam deflector patches are used. However, these approaches rely on interference between two beams from different regions at the SLM, and retrieving the phase requires fitting the observed fringes in the Fourier plane to a model function. This only gives a constant phase across each patch (as opposed to the local gradient measurement in our method which provides more information), which means the patches must be much smaller to get an accurate measurement. This requirement of smaller size reduces the amount of light that is deflected by the patches, which reduces the signal-to-noise ratio in the observed fringe pattern. Additionally, the fitting process relies on placing the camera sensor accurately in the focal plane because it requires the specific values of the parameters of the experimental optical system. The proposed method does not require such specific alignment and information.

## Conclusion

This work presents a thorough theoretical framework for a new method to simultaneously measure the amplitude and phase of the wavefront reflected from SLMs, which is further verified in experiments. The method operates by sequentially displaying square patches of a beam deflectors on the SLM and inferring the wavefront profile from the position and power of the associated spots in the Fourier plane by an iterative CITL approach. This process does not rely on fitting Zernike polynomials or using any additional optical components, is simple to implement, and is shown to be versatile. We analyze the reconstruction errors of such approach in simulations and verify the method experimentally by generating corrected holograms using the Gerchberg-Saxton algorithm with the measured phase and amplitude.

The proposed method is well suited for Fourier-plane holography and remains effective in nearby parallel planes, allowing it to correct defocus from potential misalignments. The wavefront characterization is performed in the exact alignment of the optical system as used for Fourier holography, thus removing the necessity for reconfiguring the system between experiments. Reconfiguring the optical system after measuring the aberrations may introduce new aberrations, and this issue is avoided with the proposed method. Finally, the method eliminates the need for additional optical components, such as a Shack-Hartmann wavefront sensor or high-precision reference mirrors, reducing system complexity and operational costs.

## Supplementary Information

Supplementary Information.

## Data Availability

The datasets generated and/or analyzed during the current study are available in the Apollo - University of Cambridge repository, https://www.repository.cam.ac.uk/handle/1810/389480.

## Code availability

The code used to generate the results in this work is available on GitHub, https://github.com/antoni-wojcik/hololab_wavecorr.
