# Supplementary material for: Fourier-plane wavefront and SLM aberration characterization via iterative scanning of beam deflector segments
Source: Sci Rep. 2025 Dec 23;16:3025. doi: 10.1038/s41598-025-32903-0 (PMC12827283; doi:10.1038/s41598-025-32903-0)
Supplement: Supplementary file 1 — Supplementary Information. [file 41598_2025_32903_MOESM1_ESM.pdf]

Supplementary Notes

Fourier-plane wavefront and SLM aberration characterization via iterative scanning of beam deflector segments

Antoni J. Wojcik[1,*], Dilawer Singh[1], Ayan Rakshit[1], Hannah J. Joyce[1], and Timothy D. Wilkinson[1]

[1]Electrical Engineering Division, Department of Engineering, University of Cambridge, 9 JJ Thomson Avenue, Cambridge CB3 0FA, UK
[*]ajw308@cam.ac.uk

Wavefront reconstruction

After measuring all the gradient vectors $\mathbf{g}_{mn}$ as described in the "Implementation" section of the main text, the wavefront phase $\phi_{\text{wav}}(x,y)$ can be reconstructed. The SLM surface is divided into $M \times N$ square patches, each with side length $a = S\Delta_{\text{SLM}}$, where S is the number of pixels per patch side and Δ_{SLM} is the pixel pitch. Each patch is indexed by (m,n), with $m = 0,1,\ldots,M-1$ and $n = 0,1,\ldots,N-1$, and is centered at coordinates (x_{mn}, y_{mn}). The reconstruction of $\phi_{\text{wav}}(x,y)$ proceeds in two steps:

1. Interpolate $\mathbf{g}_{mn}$ to obtain the smooth gradient $\mathbf{g}(x,y)$ over the whole SLM. This is done by building an approximate surface $f(x,y)$ using the mean values of each patch μ_{mn}, separately for the axes $g_x(x,y) = \partial\phi_{\text{wav}}/\partial x$ and $g_y(x,y) = \partial\phi_{\text{wav}}/\partial y$. For each patch (m,n), corner values $V_{m,n}, V_{m+1,n}, V_{m,n+1}, V_{m+1,n+1}$ are defined. These values are shared between neighboring patches to ensure continuity (a total of $(M+1)\times(N+1)$ values for $M \times N$ patches) and are used to compute the surface piece $f_{mn}(\tilde{x},\tilde{y})$ via bilinear interpolation:

$$f_{mn}(\tilde{x},\tilde{y}) = (1-\tilde{x})(1-\tilde{y})V_{m,n} + \tilde{x}(1-\tilde{y})V_{m+1,n} + (1-\tilde{x})\tilde{y}V_{m,n+1} + \tilde{x}\tilde{y}V_{m+1,n+1} \tag{S1}$$

where $(\tilde{x},\tilde{y}) = ((x-x_{mn})/a + 0.5, (y-y_{mn})/a + 0.5)$ are the normalized coordinates within the patch such that $\tilde{x},\tilde{y} \in [0,1)$, and $f_{mn}(\tilde{x},\tilde{y}) = 0$ outside of this range. The average value across the patch is calculated as:

$$\mu_{mn} = \iint_{\text{patch}} f_{mn}(\tilde{x},\tilde{y})\,\mathrm{d}\tilde{x}\,\mathrm{d}\tilde{y} = \frac{1}{4}\left(V_{m,n} + V_{m+1,n} + V_{m,n+1} + V_{m+1,n+1}\right) \tag{S2}$$

Since the values of μ_{mn} are known (the components of $\mathbf{g}_{mn}$ along the two axes), the problem requires solving a system of linear equations given by Eq. (S2) for each V_{mn}. It is an underdetermined system, because there are $(M+1)\times(N+1)$ unknown values of V_{mn}, but only $M \times N$ known values of μ_{mn}, and so $M \times N$ equations. The system of equations can be rewritten in the form: $B\mathbf{v} = \mu$, where $\mathbf{v}$ and μ are vectorized matrices V_{mn} and μ_{mn}, respectively, and B is a sparse matrix that encodes the numerical coefficients from Eq. (S2). The system of equations can be solved by least squares minimization: $\min_{\mathbf{v}} |B\mathbf{v} - \mu|^2$. The equation is built and solved for $\mathbf{v}$, separately for both gradient axes $g_x(x,y)$ and $g_y(x,y)$. After this, the values V_{mn} are extracted from $\mathbf{v}$, and Eq. (S1) is used to calculate $f_{mn}(\tilde{x},\tilde{y})$ at each patch, which can be stitched together at the edges to reconstruct the full $g_x(x,y)$ and $g_y(x,y)$, and so $\mathbf{g}(x,y)$.

2. Retrieve $\phi_{\text{wav}}(x,y)$ from the interpolated $\mathbf{g}(x,y)$ by solving the resultant Poisson equation obtained by applying the divergence operator to both sides of $\mathbf{g} = \nabla\phi_{\text{wav}}$[S1]:

$$\nabla\cdot\mathbf{g} = \frac{\partial g_x}{\partial x} + \frac{\partial g_y}{\partial y} = \frac{\partial^2\phi_{\text{wav}}}{\partial x^2} + \frac{\partial^2\phi_{\text{wav}}}{\partial y^2} = \nabla^2\phi_{\text{wav}} \tag{S3}$$

This equation can be solved for $\phi_{\text{wav}}(x,y)$ using Fourier transforms:

$$\begin{aligned} &\mathscr{F}\left\{\nabla^2\phi_{\text{wav}}\right\} = -(2\pi)^2(u^2+v^2)\mathscr{F}\left\{\phi_{\text{wav}}\right\} = \mathscr{F}\left\{\nabla\cdot\mathbf{g}\right\} \\ &\Rightarrow \phi_{\text{wav}}(x,y) = -\mathscr{F}^{-1}\left\{\frac{\mathscr{F}\left\{\nabla\cdot\mathbf{g}\right\}(u,v)}{(2\pi)^2(u^2+v^2)}\cdot\mathbf{1}_{(u,v)\neq(0,0)}\right\} \end{aligned} \tag{S4}$$

where $\mathbf{1}_{(u,v)\neq(0,0)}$ is an indicator function equal to 0 when $(u,v) = (0,0)$ and 1 otherwise. It appears because $(u,v) = (0,0)$ corresponds to the mean value of $\phi_{\text{wav}}(x,y)$ which can be set to zero because phase can be freely shifted by a constant. This process results in phase patterns with C^1 class smoothness.

When discretized at the SLM pixel level, discrete Fourier transforms assume periodic boundary conditions, which is unlikely for the aberrations, which in turn can introduce artifacts. To avoid this, the best we can do is assume Neumann boundary conditions (zero gradient at the edges) instead of periodic ones: $\nabla\phi_{\text{wav}} \cdot \hat{\mathbf{n}} = 0$, where $\hat{\mathbf{n}}$ is the normal vector to the boundary. This way the phase flattens out outside the boundaries of the SLM, as we do not have gradient information there.

In this case, Eq. (S3) can be solved using the eigenbasis of the Laplace operator with Neumann boundary conditions, which are cosine functions (in 1D the Poisson equation takes the form $\mathrm{d}^2\phi_{\text{wav}}/\mathrm{d}x^2 = \mathrm{d}g_x/\mathrm{d}x$, and the solution is $\phi_{\text{wav}}(x) = \sum_{k=0}^{\infty} a_k \psi_k(x)$, where a_k are the eigenvalues, and $\psi_k(x) = \cos(\pi k x/L)$ are the eigenfunctions, where L is the length of the domain and $k = 0, 1, 2, \ldots$. This can be extended to 2D by using separation of variables). This means that the solution should consist of even functions in both x and y directions, which can be achieved by extending $\mathbf{g}(x,y)$ symmetrically by reflecting it about the boundaries of the SLM (making it even), before applying the Fourier transform method described in Eq. (S4). The resulting $\phi_{\text{wav}}(x,y)$ is then cropped to the original SLM size. This approach effectively enforces Neumann boundary conditions.

Alternatively, the Poisson equation for the discretized $\mathbf{g}(x,y)$ and $\phi_{\text{wav}}(x,y)$ can be solved directly using discrete cosine transforms (DCTs)[S2,3], which inherently assume Neumann boundary conditions.

Recovering $\phi_{\text{wav}}(x,y)$ is challenging because the derivative relation $\mathbf{g} = \nabla\phi_{\text{wav}}$ inherently loses information about the constant offsets between different patches and only the mean gradients are measured. This means that small errors in gradients can cause the reconstructed phase to drift over long distances. To mitigate this, we assume a smooth phase and its gradients (C^1 class smoothness) and apply bilinear interpolation as a natural approximation under this limited information.

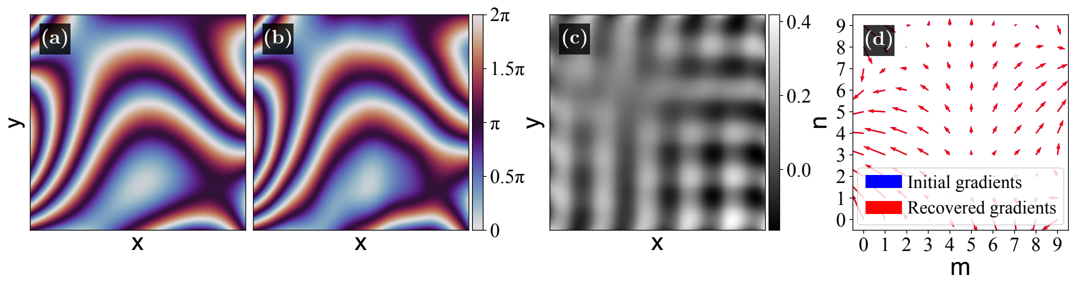

Figure S1. Reconstruction of an arbitrary phase profile using an array of mean gradients across the patches. (a), (b) Original and recovered phases, wrapped to 2π. (c) Difference between (a) and (b). (d) Initial and the recovered gradients $\mathbf{g}_{mn}$ and $\mathbf{g}_{\text{rec},mn}$ at each patch. Both sets of vectors are nearly identical, and overlap perfectly.

An example phase reconstruction of a known initial phase is shown in Fig. S1, where the initial phase is formed over $P \times Q = 1000 \times 1000$ pixels, and the size of the patch side $S = 100$ determines the number of patches to be $M \times N = 10 \times 10$. The recovered gradients shown in Fig. S1(d) are defined as $\mathbf{g}_{\text{rec},mn} = \langle \nabla\phi_{\text{wav}}(x,y) \rangle_{mn}$ - the mean gradient value of the reconstructed phase within the patch (m,n). The recovered gradients and the reconstructed phase match the initial values, demonstrating the feasibility of this approach with a coarse patch size. The difference shown in Fig. S1(c) confirms that the method avoids the accumulation of large errors over 1000 x 1000 pixels.

Reconstruction errors

The section above discusses reconstruction assuming known gradients, but does not quantify how the reconstructed pattern relates to the original phase and amplitude of the effective incident wavefront. To examine this, we simulated an SLM of 800×800 pixels, with known phase aberrations and incident amplitude, and then simulated the measurement procedure described in "Methods" in the main manuscript to measure them. In the simulation, the gradients were obtained using the iterative scanning of $M \times N$ beam deflector patches, mimicking the actual experimental process, and the virtual camera parameters are chosen so that it can resolve the entire first diffraction order. The central reference patch attempts to deflect the point to the location $(u_0, v_0) = (0.1/\Delta_{SLM}, 0.1/\Delta_{SLM})$. No tip/tilt aberrations are assumed in this section unless stated otherwise. The mean gradient is subtracted from the measured gradients to remove the bias from the gradient measurement caused by the central reference patch in the scanning process. Then, the effective wavefront amplitude and phase are estimated. This approach allows us to study the reconstruction accuracy and to identify the errors accumulated throughout the procedure.

Fig. S2 compares the true amplitude and phase to their measured counterparts as the patch grid increases from $M \times N = 5 \times 5$ to 10×10 and 20×20, with patch sizes $S =$ 160, 80, and 40 pixels. Both the measured amplitude and phase resemble the

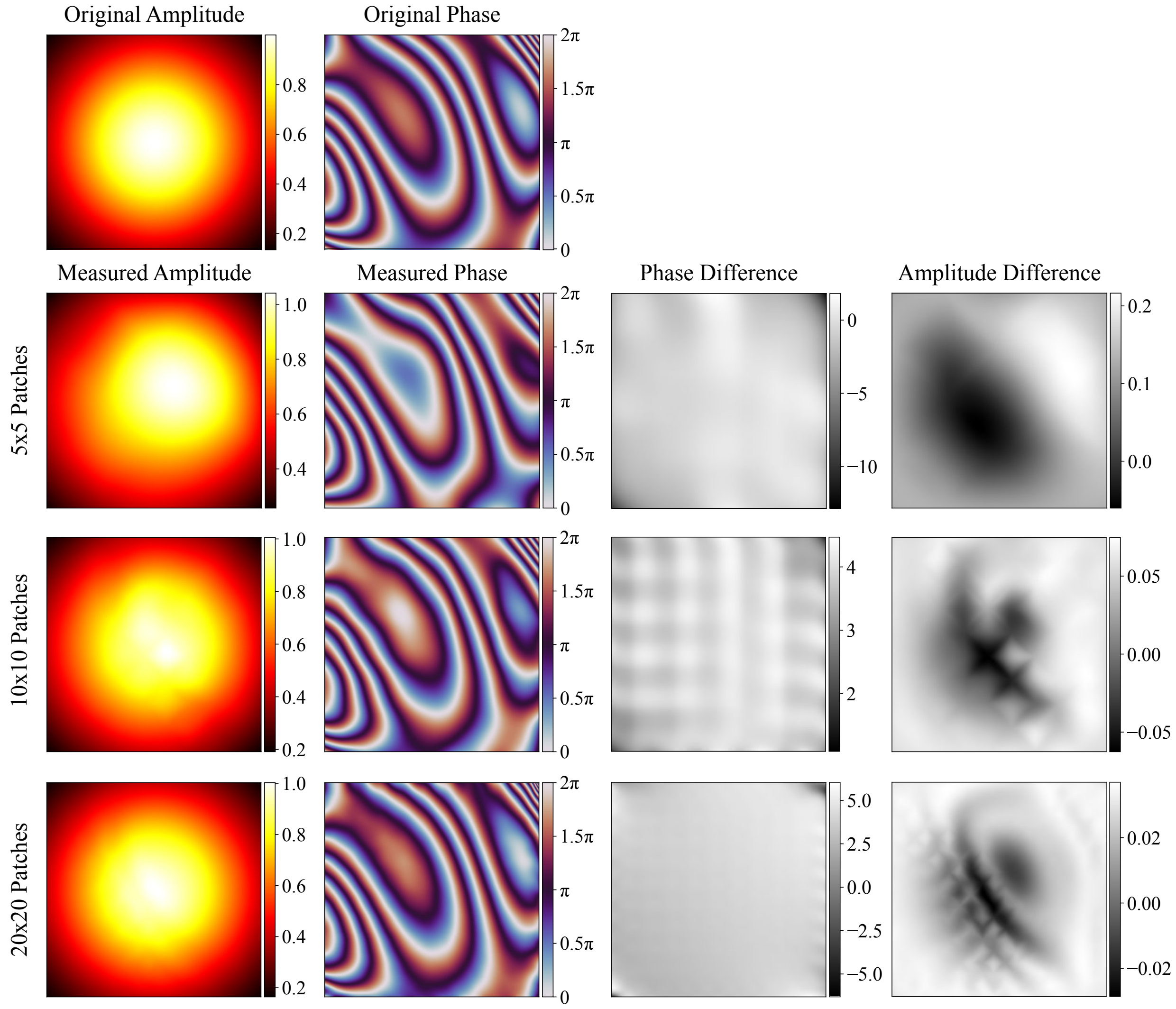

Figure S2. Simulated amplitude and phase reconstruction errors with different numbers of patches.

ground truth, and the error reduces with more patches. At the lowest sampling, the amplitude distribution appears slightly shifted, and while the overall errors in phase exceed 2π radians, this large difference is contained to the corners of the SLM, and most of the error is significantly smaller in the remaining area. At coarse sampling, large regions of phase and amplitude are effectively averaged into the local gradient and amplitude values.

A further source of error arises from this per-patch averaging, which can be seen from the diffraction integral. Defining $\mathbf{x} = (x,y)$ and $\mathbf{u} = (u,v)$, and the true amplitude $A_{\mathrm{wav}}(\mathbf{x})$ and phase $\phi_{\mathrm{wav}}(\mathbf{x})$, and the displayed hologram $\phi(\mathbf{x})$, we can write:

$$U(\mathbf{u}) = \iint_{\mathbb{R}^2} E(\mathbf{x}) e^{-2\pi j \mathbf{x}\cdot\mathbf{u}} \,\mathrm{d}x\,\mathrm{d}y = \iint_{\mathbb{R}^2} A_{\mathrm{wav}}(\mathbf{x}) e^{j(\phi_{\mathrm{wav}}(\mathbf{x})+\phi(\mathbf{x}))} e^{-2\pi j \mathbf{x}\cdot\mathbf{u}} \,\mathrm{d}x\,\mathrm{d}y. \tag{S5}$$

Assuming that $\phi(\mathbf{x})$ contains a single beam deflector patch (m,n) with the measured gradient $\tilde{\mathbf{g}}_{mn}$, centered at $\mathbf{x}_{mn}$, define $\mathbf{r}_{mn} = \mathbf{x} - \mathbf{x}_{mn}$ and expand $\phi_{\mathrm{wav}}(\mathbf{x}) = \phi_{\mathrm{wav}}(\mathbf{x}_{mn}) + \nabla\phi_{\mathrm{wav}}(\mathbf{x}_{mn}) \cdot \mathbf{r}_{mn} + {}^{1}\!/_{2}\,\mathbf{r}_{mn}^{T}\mathbf{H}\mathbf{r}_{mn} + \ldots$, where $\mathbf{H}$ is the Hessian. Since $\nabla\phi_{\mathrm{wav}}(\mathbf{x}_{mn}) = \mathbf{g}_{mn}$ and $\phi(\mathbf{x}) = \tilde{\mathbf{g}}_{mn} \cdot \mathbf{x} = -\mathbf{g}_{mn} \cdot \mathbf{x}$, we obtain the residual phase $\phi_R(\mathbf{x}) = \phi_{\mathrm{wav}}(\mathbf{x}) + \phi(\mathbf{x}) = {}^{1}\!/_{2}\,\mathbf{r}_{mn}^{T}\mathbf{H}\mathbf{r}_{mn} + \ldots$. This $\phi_R(\mathbf{x})$ within the patch behaves as a local aberration and cannot be captured by the proposed method for a given patch size S. In strong cases, it may bias the measured gradient so that $\tilde{\mathbf{g}}_{mn} \neq -\mathbf{g}_{mn}$. Figs. S3(a)-(c) show how the shape of the deflected spot changes as the patch size gets smaller. For large patch sizes, the observed spot is smaller and more aberrated because $\phi_R(\mathbf{x})$ is larger and more relevant. Reducing S is the only way with this approach to make $\phi_R(\mathbf{x})$ negligible at sufficiently small scales.

Figs. S3(d)-(f) show a spot with increasing aberrations: in (d) the gradient approximation begins to fail, and by (f) it has fully broken down. This occurs when the aberrations vary at spatial frequencies above the Nyquist limit set by the patch sampling. With patches of size $S \times S$, the sampling frequency is $1/S$, so the method can only detect phase and amplitude

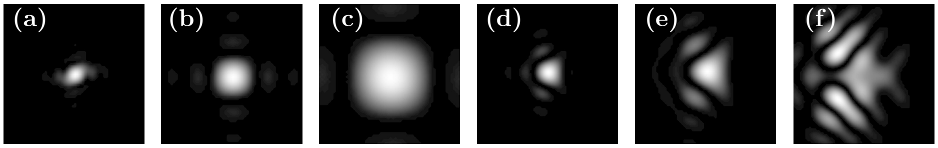

Figure S3. Spot shapes with when (a)-(c) the number of patches is changed from 5×5, across 10×10, to 20×20, and (d)-(f) for 10×10 patch grid but with severe aberrations with spatial frequencies of $0.8\times$, $1\times$, and $1.6\times$ the Nyquist frequency.

variations up to the Nyquist frequency $1/(2S)$.

Fig. S4 illustrates this behavior for a grid of $M = N = 10$ patches ($S = 160$). When $\phi_{\text{wav}}(\mathbf{x})$ varies well below the Nyquist limit (2 periods/SLM), reconstruction errors are small. Just below the limit (4 periods/SLM), errors become noticeable but the overall structure remains correct. At the limit (5 periods/SLM), aliasing starts to appear, and far above it (8 periods/SLM), the reconstruction breaks down entirely. The 8-period case was selected to produce perfect aliasing with the 2-period case (same distance from the 5-period Nyquist frequency), which is why their recovered phases look similar.

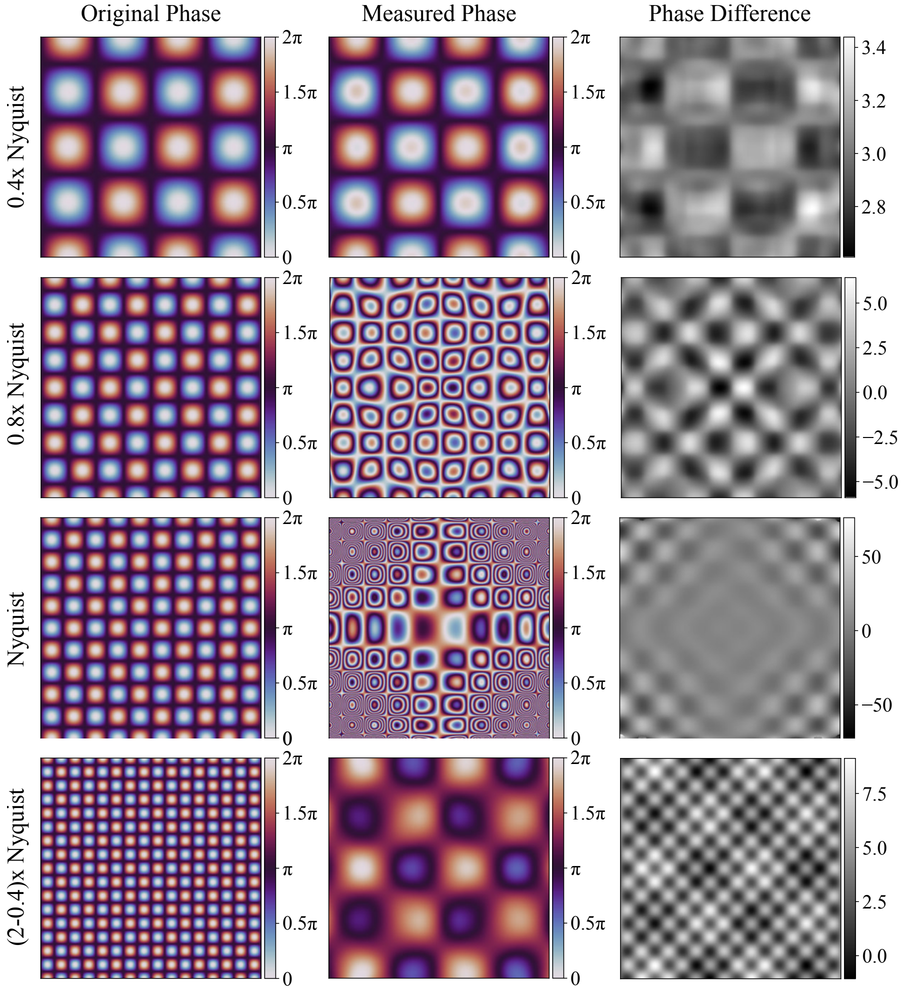

Figure S4. Errors as the aberration variation approaches and surpasses the Nyquist frequency (5×5 patches).

In practice, reconstruction errors are further increased by experimental factors such as camera shot noise (especially when spots are faint in low-amplitude regions of the incident beam), pixel saturation, or spot sizes that are too large or too small for reliable estimation of the center of mass of the spot during the scanning process.

Even without significant reconstruction errors, the method is inherently biased to the overall tip/tilt aberrations (as are the interferometric aberration measurement techniques). This is the case because the central reference patch can also have its own local tip and tilt aberrations. Subtracting the mean gradient does not resolve this, because the absolute gradient information is

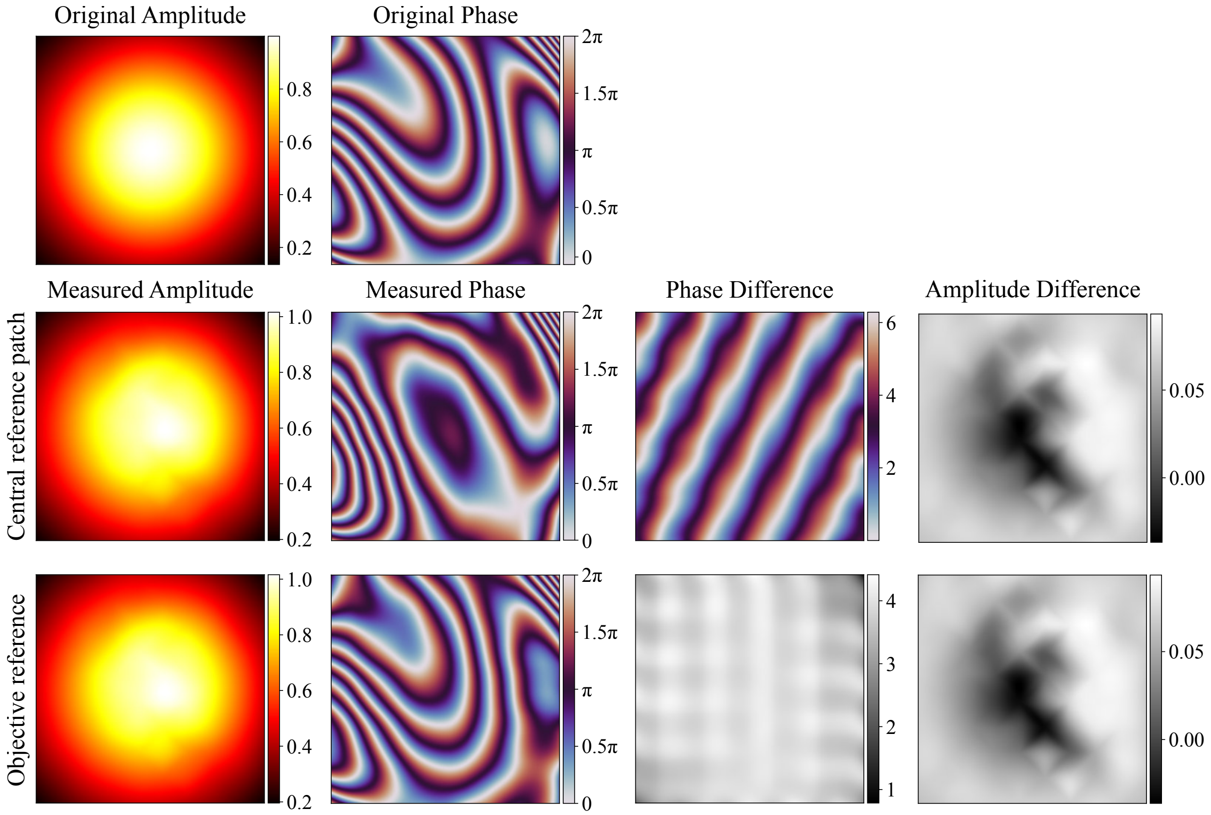

Figure S5. Emergence of constant tip/tilt bias when using the central reference patch ($M \times N = 10 \times 10$ patches). The bias disappears when the objective reference point is used.

lost, as illustrated in Fig. S5, where the aberrations include a global tip/tilt component. This is usually not a major issue, since such aberrations only shift the image by a small amount in the focal plane and can be compensated by pre-shifting the target pattern. However, if the true absolute tip/tilt correction is required, the spot positions must be referenced to an objective point other than the central patch, that is the ideal theoretical location of that point if no aberrations are present: $(f\lambda u_0, f\lambda v_0)$ (see bottom of Fig. S5). In practice, locating this point accurately under experimental conditions can be challenging, but it may be determined in its relation to the zeroth order, the location of which is not typically affected by aberrations.

Other experiments

The main manuscript introduces measurement of the effective incident wavefront, but only shows measurements performed for a Fourier holographic system in the off-axis configuration. Here, we show the results for the on-axis configuration shown in Fig. S6, with different SLM models, lenses, and laser wavelengths.

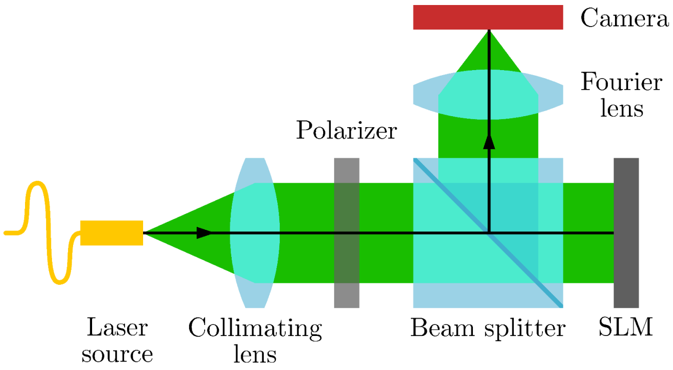

Figure S6. Off-axis holographic projector configuration.

Santec SLM-250

The optical system uses a laser source with wavelength $\lambda = 405\,\mathrm{nm}$, a Fourier lens with focal length $f = 10\,\mathrm{cm}$, and a 1920×1200-pixel Santec SLM-250 (UV-resistant) for phase-only modulation. The effective wavefront is measured using $M \times N = 16 \times 10$ patches of size $S = 120$, with the measurement process taking an average of 15 min. The measured amplitude and phase profiles are shown in Fig. S7 and are tested on an example projected pattern shown in Fig. S8 (average of 690 independently generated holograms). The phase pattern in Fig. S7(d) shows a strong quadratic component, suggesting a strong defocus correction.

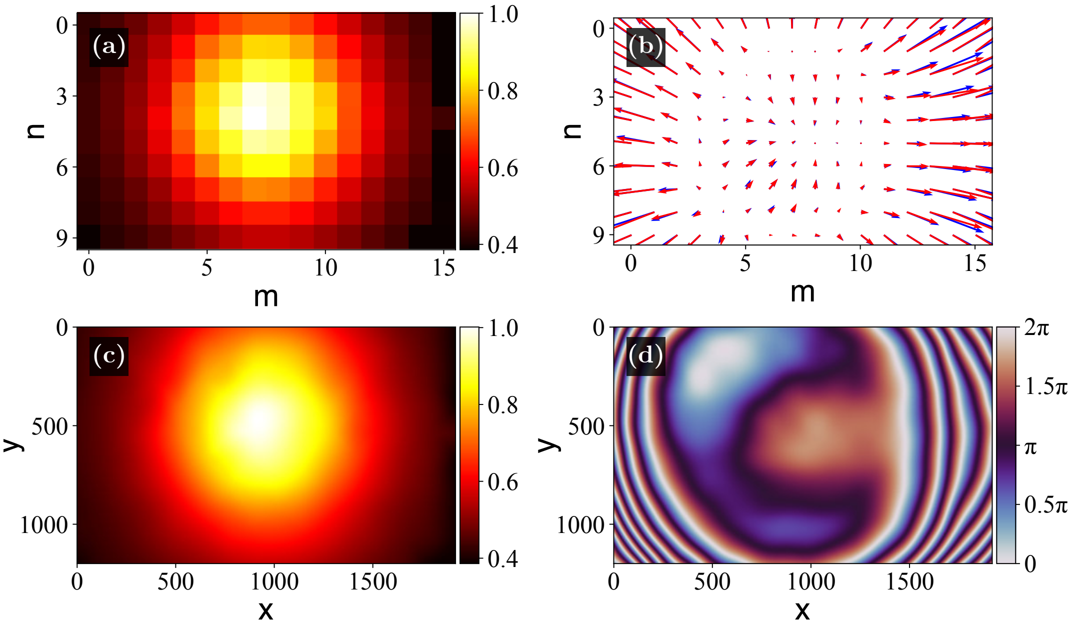

Figure S7. (a), (b) Measured normalized amplitudes and gradients at each patch. (c), (d) Amplitude and phase across the area of the SLM recovered from the patch data in (a) and (b).

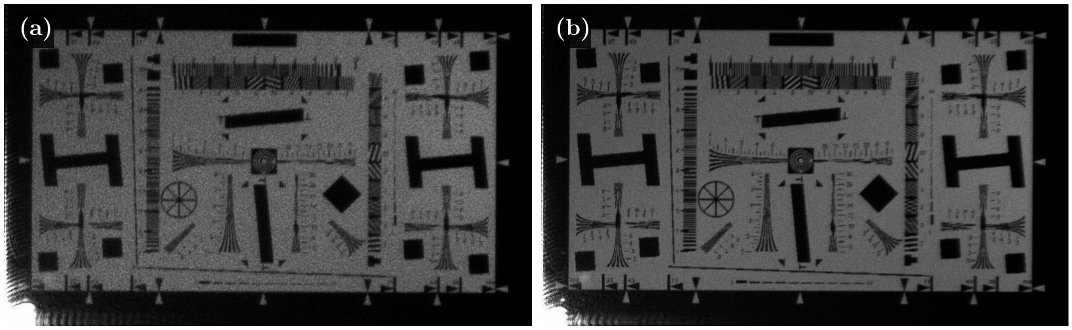

Figure S8. Hologram projection (a) without any correction, and (b) with the measured wavefront correction.

Holoeye LUNA

The optical system uses a laser source with wavelength $\lambda = 635\,\mathrm{nm}$, a Fourier lens with focal length $f = 4.5\,\mathrm{cm}$, and a 1920×1080-pixel Holoeye LUNA phase-only SLM. The effective wavefront is measured using $M \times N = 16 \times 9$ patches of size $S = 120$. The wavefront characterization takes an average of 50 s thanks to the fast response time of the SLM: we limited the delay between displaying the hologram and camera capture to 100 ms, and the scanning process typically requires 3 iterative measurements per patch. The measured amplitude and phase profiles are shown in Fig. S9 and are tested on an example projected pattern shown in Fig. S10. The holograms were generated using the CGH process described in the main manuscript.

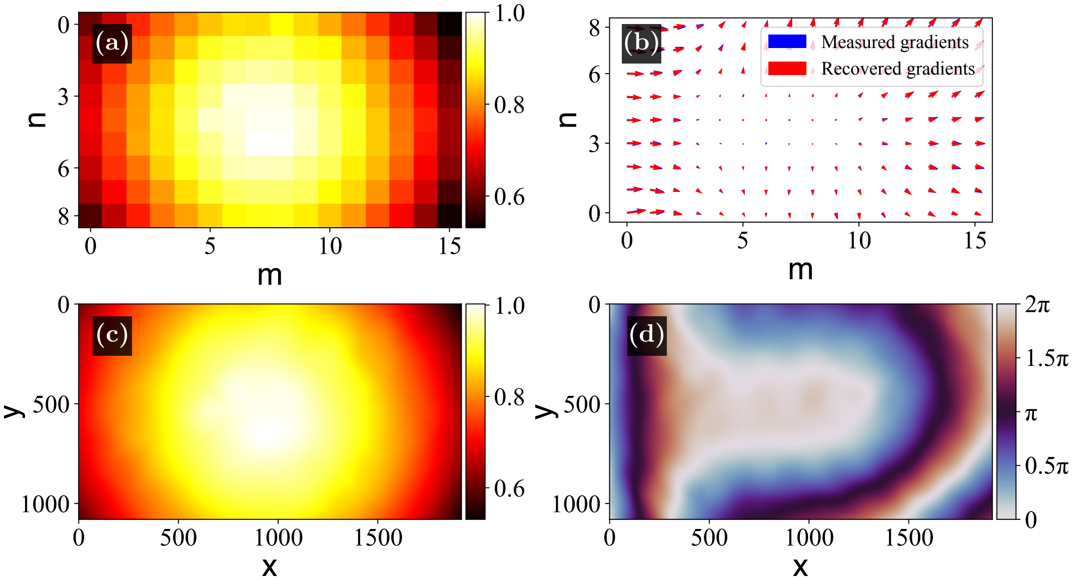

Figure S9. (a), (b) Measured normalized amplitudes and gradients at each patch. (c), (d) Amplitude and phase across the area of the SLM recovered from the patch data in (a) and (b).

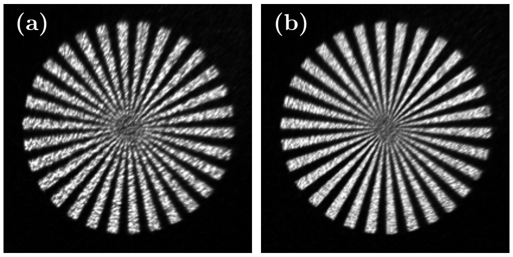

Figure S10. Hologram projection (a) without any correction, and (b) with the measured wavefront correction.

References

S1. Bhat, P., Curless, B., Cohen, M. & Zitnick, C. L. Fourier analysis of the 2d screened poisson equation for gradient domain problems. In Forsyth, D., Torr, P. & Zisserman, A. (eds.) *Computer Vision – ECCV 2008*, 114–128 (Springer Berlin Heidelberg, Berlin, Heidelberg, 2008).

S2. Schumann, U. & Sweet, R. A. Fast fourier transforms for direct solution of poisson's equation with staggered boundary conditions. *J. Comput. Phys.* **75**, 123–137, DOI: https://doi.org/10.1016/0021-9991(88)90102-7 (1988).

S3. Strang, G. The discrete cosine transform. *SIAM Rev.* **41**, 135–147, DOI: 10.1137/S0036144598336745 (1999).
